# Mid-term results of Miller−Galante unicompartmental knee replacement for medial compartment knee osteoarthritis

**DOI:** 10.1007/s10195-015-0385-4

**Published:** 2015-11-14

**Authors:** Hemanth Kumar Venkatesh, S. S. Maheswaran

**Affiliations:** 1 Department of Orthopedics, Basildon and Thurrock University Hospital, Nethermayne, Basildon, Essex SS165NL UK; 2 Department of Orthopedics, University Hospital of North Tees & Hartlepool, Stockton On Tees, TS19 8PE UK

**Keywords:** Arthritis, Medial compartment knee, Fixed bearing, UKR

## Abstract

**Background:**

The purpose of this study is to analyse and report the mid-term results of 175 unicompartmental knee replacement (UKR) procedures performed for medial compartment knee arthritis from January 2001 to January 2010.

**Materials and methods:**

The cohort participants were selected after stringent inclusion criteria and the average follow-up was 5.6 years (range 2–10 years). The fixed-bearing UKR procedure was carried out on all patients.

**Results:**

The pre-operative mean knee range of movement improved from 100° ± 11.3° to 118.3° ± 12° (*p* value <0.001). The pre-operative mean Knee Society (KS) knee and functional score improved from 47 ± 5.5 and 55.1 ± 4.6 to 91.8 ± 9.2 and 92 ± 10.1 (*p* value <0.001), respectively. The revision rate of the cohort was 4 % (seven knees) and implant survival rate was 96 % at the end of 10 years; 87 % of the cohort were satisfied with the procedure and had a normal gait pattern. In this study, there was no statistical difference between groups with a body mass index (BMI) ≤30 kg/m^2^ and those with a BMI ≥30 kg/m^2^, and between groups aged ≤55 years and those aged ≥55 years, in clinical and functional outcome following UKR.

**Conclusion:**

This study confirms that fixed-bearing UKR gives excellent results in patients with medial compartment knee arthritis who comply with the inclusion criteria. Age and BMI were not considered to influence the clinical and functional outcomes.

Level of evidence-III.

## Introduction

The degenerative changes in idiopathic osteoarthritis of the knee begin in the medial compartment in 80–90 % of patients [1–3]. This has given rise to the rationale for the treatment of only one compartment, either with a high tibial osteotomy (HTO) or a unicompartmental knee replacement (UKR). Improved mid- and long-term results of UKR, comparable with the excellent and well-known results after total knee replacement (TKR), have contributed to the use of UKR on younger, active, and obese populations [4].

For the past 20 years, the overall results of UKR have been promising, and this procedure is especially appropriate for anteromedial osteoarthritis of the knee [5–7]. UKR is less invasive, causes less blood loss, and preserves the bone stock and almost normal knee kinematics in comparison with TKR [8–11].

The purpose of this study was to evaluate the mid-term results of the fixed-bearing metal-backed Miller−Galante prosthesis implanted in 148 patients with medial compartmental osteoarthritis and also to evaluate the functional outcome in groups aged ≤55 years and ≥55 years, and in groups with a body mass index (BMI) ≤30 kg/m^2^ and ≥30 kg/m^2^. Our hypothesis was that UKR surgery improves the clinical and functional outcome in patients with medial compartment arthritis of the knee joint. The criteria for selecting patients for UKR were thoroughly analysed since much controversy exists about the correct indications for this procedure.

## Materials and methods

The research population comprised 148 patients with 175 primary UKRs at the University Hospital of North Tees and Hartlepool Trust, UK between January 2001 and January 2010.

**Fig. 1:**
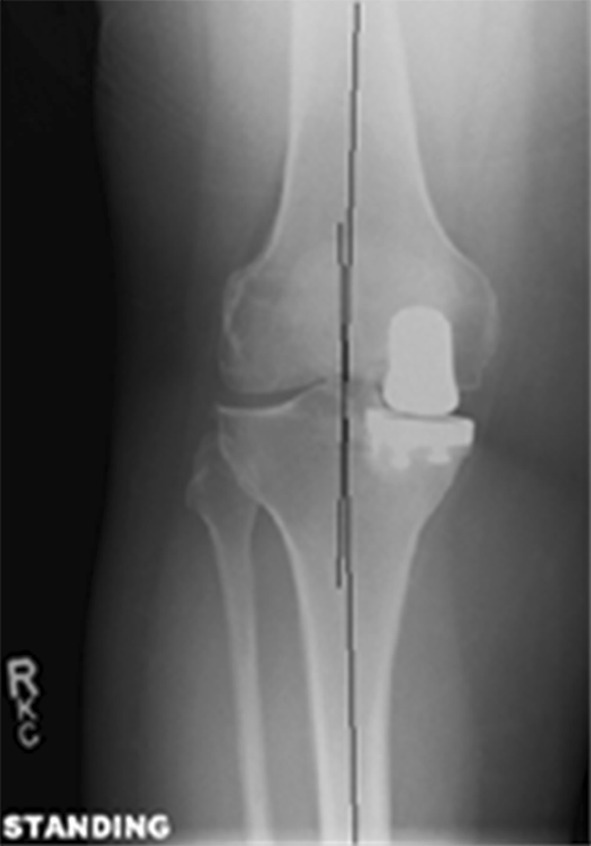
Post UKR, alignment angle AP view

**Fig. 2:**
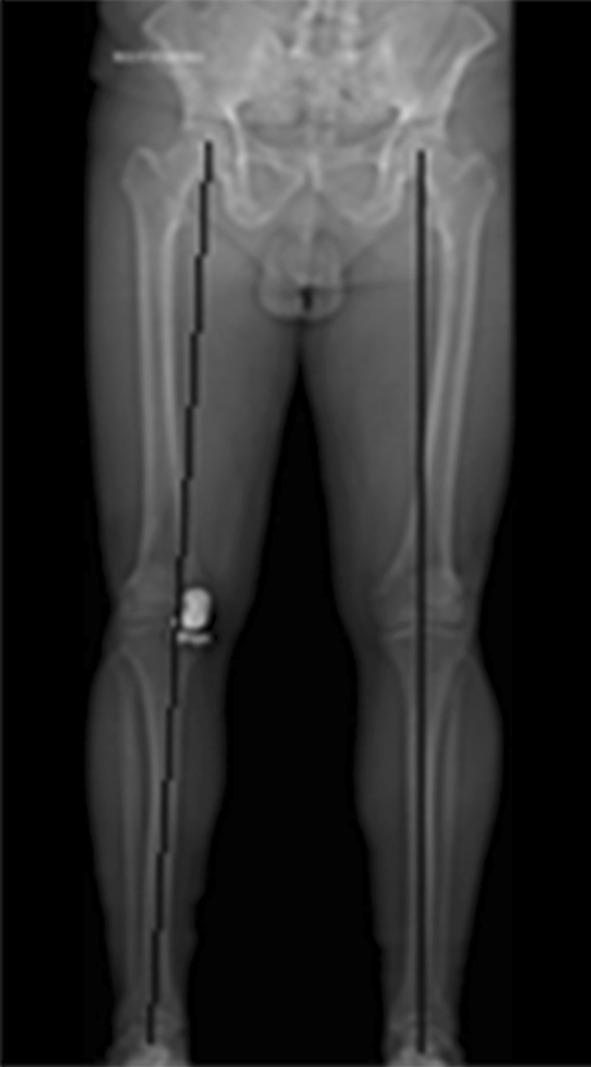
Orthogonal view post UKR

Patients were selected after thorough clinical and radiological evaluation, and only those with medial compartment disease were selected after fulfilment of inclusion and exclusion criteria. Inclusion criteria were patients aged ≥40 years, BMI <40 kg/m^2^, no pain at rest, medial compartment osteoarthritis (Ahlbäck radiological grades 3 or 4), intact anterior and posterior cruciate ligament (ACL and PCL, respectively), flexion deformity ≤10°, correctable varus deformity ≤15°, and minimum 90° of knee flexion (Figs. 1, 2).

Patients with active or suspected infection in the knee, inflammatory arthritis (rheumatoid arthritis, gout, psoriatic arthritis), a previous history of HTO, post-traumatic arthritis, advanced osteoarthritis involving the lateral compartment and lateral facet arthritis of the patellafemoral (PF) joint were excluded from the study.

Complete radiological assessment was carried out before surgery to assess the degree of deformity, and severity of arthritis [3]. Standard weight-bearing anteroposterior (AP) and lateral radiographs of the knee joint were used in all patients. Varus and valgus stress views of the knee joint were taken to confirm the presence of full-thickness articular cartilage in the lateral compartment. A skyline view of the patella was used to assess the PF joint status. Mechanical axis and degree of varus deformity were estimated by orthogonal views.

All the surgical procedures were performed by the same senior surgeon (SSM), with metal-backed cemented-fixed Miller−Galante (Zimmer, Warsaw, IN, USA) UKRs in 175 knees. Of 175 primary UKRs, osteoarthritis of the medial compartment knee was common and involved 147 patients with 174 knee joints. Arthritis secondary to osteonecrosis of the medial femoral condyle was noted in one patient. Bilateral staged UKR was performed in 27 patients.

Of 175 patients, 72 (41 %) had undergone previous knee surgeries—67 patients had undergone arthroscopic debridement of the joint including partial medial meniscectomy, four patients had undergone open meniscectomy and one patient had undergone arthroscopic ACL reconstruction in the past prior to index surgery.

The surgical findings and the status of the PF joint cartilage and lateral compartment cartilage were recorded from surgical notes. Intra-operative and post-operative complications such as fracture, infection, bleeding and re-surgery were also recorded.

### Surgical procedure

All the procedures were performed under regional anaesthesia. Standard medial parapatellar approach from the upper pole of the patella to 1–2 cm distal to the joint line, proximal (1–2 cm) to the tibial tuberosity was performed. The patella was never dislocated, only lateralized. The tibial resection was performed with an extra medullary guide with a slope of 5°. Based on the tibial resection, the distal femur was resected through a ‘spacer block’ that allowed a cut parallel (in extension) to the tibial cut. The femoral cutting block was then positioned for the posterior femoral and oblique resections. The trial components were positioned, and dynamic tests were performed to choose the polyethylene thickness. The flexion and extension gaps were assessed, trying to obtain approximately 2 mm of laxity in both positions. Soft-tissue release was not necessary in any of the cases. Finally, the definitive components were fixed with Palacos^®^ cement (Zimmer). The thickness of the polyethylene ranged from 8−12 mm. A periarticular injection with local anaesthetic was given before implantation. An immediate full weight-bearing rehabilitation protocol was used for all the patients. The patients also received routine thromboprophylaxis with low-molecular-weight heparin for 2 weeks post-operatively.

Clinical and functional evaluations were performed during post-operative follow-up at regular intervals of 3 months, 6 months, and 1 year and Knee Society Scores (KSS) were used to compare the overall functional and clinical results. Post-operative radiographs were assessed for alignment of the components, correction of deformity, and signs of loosening of the components. Clinical and radiological assessments were performed at the final follow-up. A patient satisfaction survey was included at the final follow-up based on the ability to perform daily living activities and no standard scoring systems were used for the assessment. The results were rated as satisfactory, excellent and not satisfactory.

Failure of the surgery was defined as the revision of UKR to TKR due to any cause such as loosening of components, infection, pain or any other indications.

### Statistical analysis

Data were entered into Microsoft Excel spread sheets and statistical analysis performed using SPSS software (SPSS Inc. version 18.0). All the scale variables were tested for normality using Kolmogorov−Smirnov test. Patient demographics were described using means, standard deviations, and ranges. The independent *t*-test was used to compare KSS clinical and functional outcomes. The level for statistical significance was <5 %, i.e., the probability that the difference measured could have been due to chance was <5 % (*p* ≤ 0.05). Paired data were analysed using a paired *t* test. Levene’s test was used to statistically test for equality of variances.

Implant survivorship was calculated by constructing survivorship tables and Kaplan–Meier survivorship analysis with 95 % confidence intervals. Revision of UKR to TKR is the final determinant of survivorship.

## Results

Of the 148 patients (86; 57.1 % male and 62; 42.1 % female), 89 knees were replaced on the right side and 86 on the left side and 27 patients underwent bilateral UKR surgery. The average age at the time of index surgery was 61.7 years (range 44–80 years), with a mean age of 62.7 years for males and 60.3 years for females. The mean BMI of the cohort at the time of index surgery was 29.2 kg/m^2^ (range 21–38 kg/m^2^); the mean BMI for males was 29  and 29.4 kg/m^2^ for females. The average follow-up of the cohort was 5.6 years (range 2–10 years). The mean length of stay in hospital following index surgery was 2.5 days (range 2–4 days) (Table 1).

**Table 1:** Demographic statistics

	*N*	Minimum	Maximum	Mean		Standard deviation
	Statistic	Statistic	Statistic	Statistic	Standard error	Statistic
Age	175	44	80	61.71	0.651	8.617
Follow-up (months)	175	22	129	63.61	1.992	26.270
BMI	175	21	38	29.19	0.286	3.781
LOS (days)	175	2	4	2.59	0.148	1.723
Pre-operative ROM	175	80	135	110.98	0.956	11.630
Post-operative ROM	175	60	135	117.34	0.997	12.287

*BMI* Body mass index, *LOS* length of stay, *ROM* range of movement

**Fig. 3:**
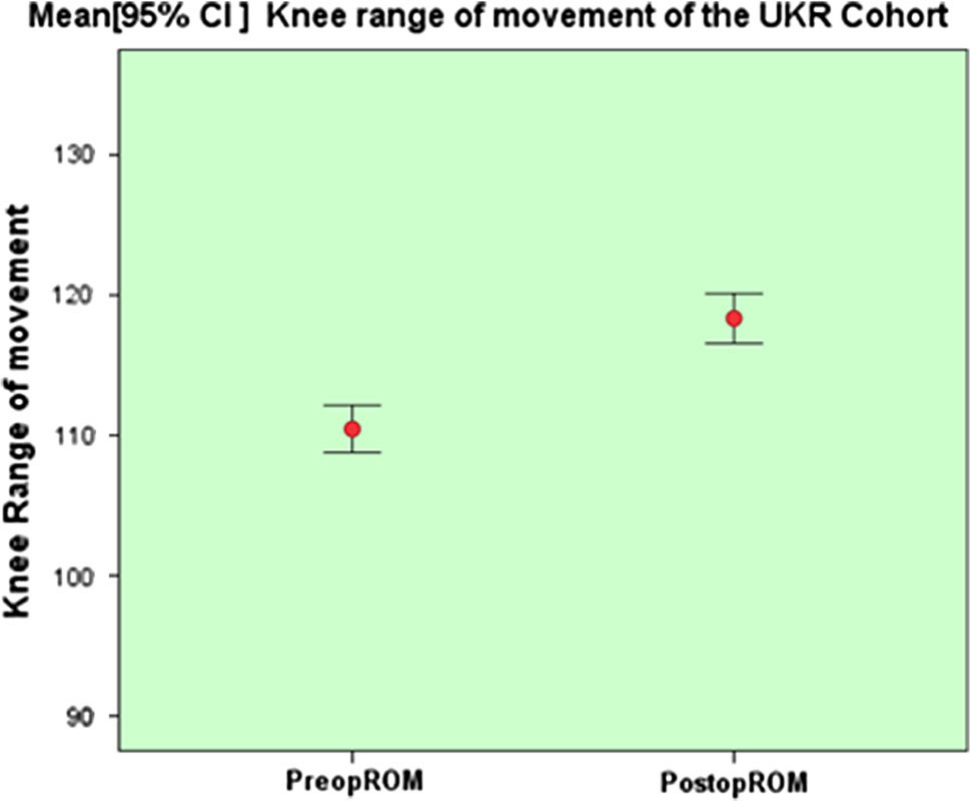
Error bar graph illustrating the knee range of movement of the cohort

The mean pre-operative knee range of movement (ROM) of the 175 UKRs improved from 110.5° (range 80°–135°) to 118.3° (range 60–135°) at the final follow-up. The mean difference was −7.9 with 95 % CI (−10.14, −5.4), *p* value <0.001 (Fig. 3).

**Fig. 4:**
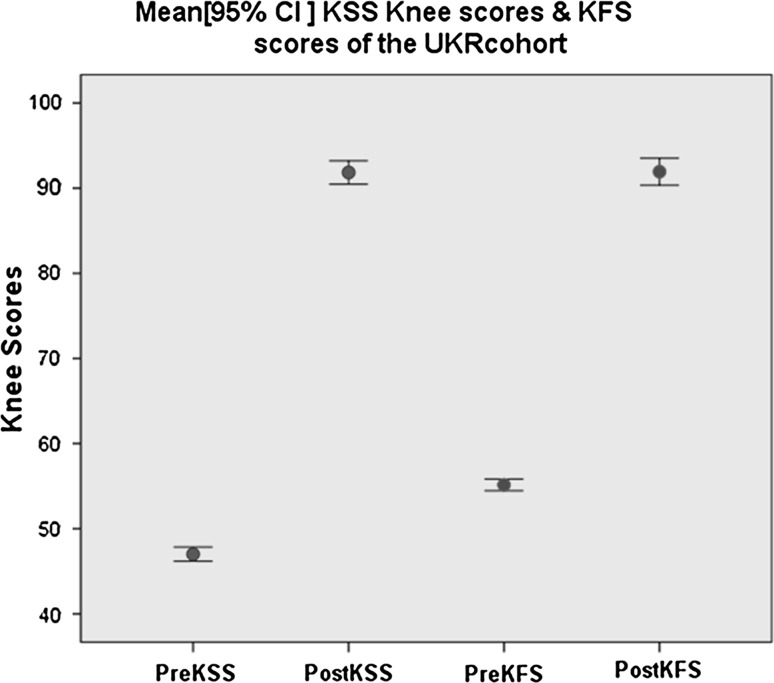
Error bar chart illustrating the knee scores of the cohort

The mean pre-operative KS knee score of the cohort improved from 47 (range 34–62) to 91.8 (range 51–100) at the final follow-up. The mean difference was −44.83 with 95 % CI (−46.44, −43.23), *p* value <0.001. The mean pre-operative KS functional score of the cohort improved from 55.1 (range 45–65) to 92 (range 55–100) at the final follow-up. The mean difference was −36.90 with 95 % CI (−38.5, −35), *p* value <0.001 (Fig. 4).

The independent *t* test found no statistical difference in knee clinical and functional scores between males and females.

The mean BMI of the cohort was 29.2 kg/m^2^ (range 21–38 kg/m^2^). The sample size with BMI ≤30 kg/m^2^ was 117 (72 male, 55 female), and the sample size with BMI ≥30 kg/m^2^ group was 58 (28 male, 30 female).

Mean pre-operative KS knee scores were 47.4 for BMI ≤30 kg/m^2^ and 46.2 for BMI >30 kg/m^2^. The mean difference was 1.2 with 95 % CI (−0.55, 2.96), *p* value = 0.832. The mean pre-operative knee functional scores were 55.3 for BMI ≤30 kg/m^2^ and 54.9 for BMI >30 kg/m^2^. The mean difference was 0.35 with 95 % CI (−1.12, 1.82), *p* value = 0.620.

Mean post-operative KS knee scores at recent follow-up for BMI ≤30 kg/m^2^ was 91.6 and 92.4 for BMI >30 kg/m^2^. The mean difference was −0.85 with 95 % CI (−3.77, 2.06), *p* value = 0.539. Mean post-operative knee functional scores at recent follow-up for BMI ≤30 kg/m^2^ were 91.6 and 92.7 for BMI >30 kg/m^2^. The mean difference was −1.12 with 95 % CI (−4.48, 2.23), *p* value = 0.703.

In the BMI ≤30 and ≥30 kg/m^2^ groups, there was no statistically significant difference in KS clinical scores, functional scores and knee ROM scores, (*p* value >0.05) (Table 2).

**Table 2:** Statistics for BMI ≥30 and ≤30 kg/m^2^ groups

	BMI-kg/m^2^	*N*	Mean	Standard deviation	Standard error mean
Pre-operative ROM	≤30	117	111.89	12.089	1.191
	≥30	58	108.89	10.329	1.540
Post-operative ROM	≤30	117	117.88	12.149	1.180
	≥30	58	116.00	12.774	1.904
Pre-operative KSS	≤30	117	49.00	5.894	0.672
	≥30	58	49.27	5.601	1.023
Post-operative KSS	≤30	117	91.94	9.830	0.955
	≥30	58	92.96	7.992	1.178
Pre-operative KFS	≤30	117	55.58	5.754	0.651
	≥30	58	55.00	4.355	0.795
Post-operative KFS	≤30	117	91.21	12.006	1.166
	≥30	58	91.98	10.012	1.476

*BMI* Body mass index, *ROM* range of movement, *KSS* knee society score, *KFS* knee functional score

The sample size for the group aged ≤55 years was 38 (17 male, 21 female). The sample size for the group aged ≥55 years was 137 (83 male, 54 female).

Mean pre-operative KS knee score was 46.4 for the group aged ≤55 years and 47.2 for the group aged ≥55 years. The mean difference was −0.77 with 95 % CI (−2.70, 1.17), *p* value = 0.809. Mean post-operative KS knee score was 92.2 for the group aged ≤55 years and 91.7 for the group aged ≥55 years. The mean difference was 0.42 with 95 % CI (−2.79, 3.63), *p* value = 0.539.

Mean pre-operative knee function score was 54.4 for the group aged ≤55 years and 55.4 for the group aged ≥55 years. The mean difference was −0.94 with 95 % CI (−2.55, 0.66) *p* value = 0.285. Mean post-operative knee function score was 91.3 for the group aged ≤55 years and 92.1 for the group aged ≥55 years. The mean difference was −0.83 with 95 % CI (−4.53, 2.88), *p* value = 0.455.

This study infers no statistical significant difference in KS clinical and functional outcomes between two age groups (*p* value >0.05) (Table 3).

**Table 3:** Statistics for groups aged ≥55 years/≤55 years

	Age	*N*	Mean	Standard deviation	Standard error mean
Pre-operative KSS	≤55	38	49.37	5.718	1.312
	≥55	137	49.01	5.834	0.622
Post-operative KSS	≤55	38	91.38	8.988	1.541
	≥55	137	92.50	9.407	0.866
Pre-operative KFS	≤55	38	54.21	4.791	1.099
	≥55	137	55.67	5.497	0.583
Post-operative KFS	≤55	38	90.15	12.522	2.147
	≥55	137	91.81	11.101	1.022
Pre-operative ROM	≤55	38	112.34	12.047	2.130
	≥55	137	110.60	11.537	1.071
Post-operative ROM	≤55	38	115.16	14.783	2.613
	≥55	137	117.90	11.581	1.062

*KSS* Knee society score, *KFS* knee functional score, *ROM* range of movement

### Patient satisfaction

At the latest follow-up, 45 % of the patients were enthusiastic regarding the procedure and 42 % patients were satisfied with the results. Twelve patients underwent TKR for the opposite side. They were very satisfied with the UKR knee outcome and preferred UKR over TKR. Ten percent of the patients were not satisfied with the procedure and 3 % of the patients did not reply.

### Radiographic results

The average pre-operative varus deformity was 7° (range 2°–14°) measured on orthogonal X-ray. The average post-operative alignment was 3° (range neutral to 5°). The average alignment at recent follow-up was 4° (range 2°–8°), and there were no signs of progression of arthritis in the lateral compartment in the cohort at the last follow-up X-ray. Medial PF joint arthritis was noted in 60 % (105 patients) of the cohort and there was no progression in PF joint arthritis at the recent follow-up X-ray. The average pre-operative grade of PF joint arthritis was Grade 2, which involved the medial facet more commonly than the trochlear groove (range grade 1–3). This was consistent with surgical findings.

### Complications

There were no significant complications per-operatively or post-operatively such as fractures, deep vein thrombosis, deep infection, and progression of arthritis in the opposite compartment. Six patients developed superficial infections post-operatively and were managed with oral antibiotics. One patient developed chronic regional pain syndrome post-operatively and was managed with medical treatment. None of the patient had any significant blood loss during the procedure or required blood transfusion post-operatively.

### Revisions

Four patients (2.28 %) underwent revision surgery to TKR because of unexplained pain. The clinical, biochemical and radiological investigation including computed tomography (CT) scan failed to identify the source of the pain. The average period for revision surgery was 31.7 months (range 19–54 months) from the time of index surgery. There was no marked improvement in the KS knee score and functional score in these patients. The average KS knee and functional scores were 59.5 (range 55–63) and 66.25 (range 60–70), respectively.

Two patients (1.14 %) underwent revision surgery to TKR at 5 and 7 years following index surgery due to tibial component loosening, which was demonstrated by CT scan without significant osteolysis.

One patient (0.57 %) had revision surgery to TKR because of polyethylene wear, 9.6 years following index surgery.

In the cohort of BMI ≤30 kg/m^2^, the failure rate was 4.27 % (five knees) and the main factor for failure was unexplained pain in 1.70 % (two knees), loosening of component 1.70 % (two knees), and polyethylene wear 0.85 % (one knee). In the cohort of BMI ≥30 kg/m^2^, the failure rate was 3.44 % (two knees) and the factor for failure was unexplained pain.

### Survival analysis

**Fig. 5:**
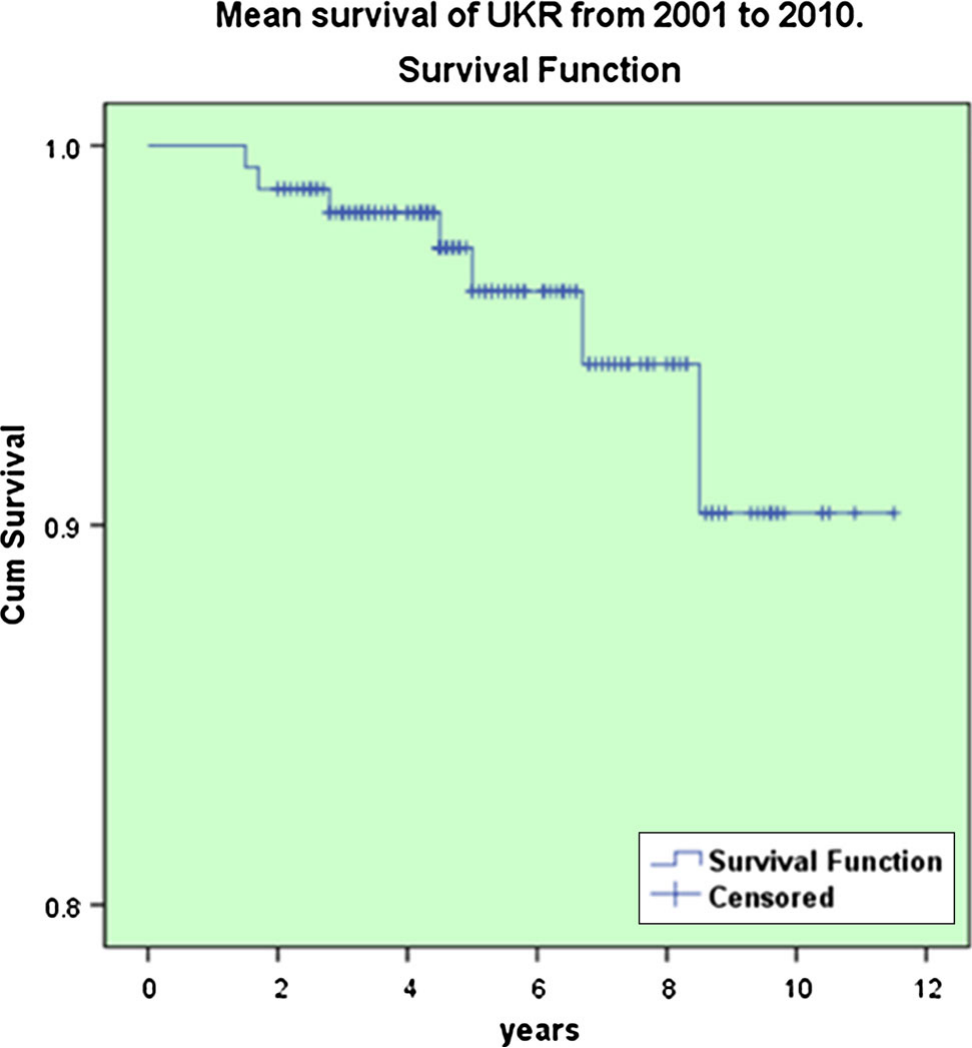
Illustrating Kaplan−Meier implant survival

Implant survival was calculated by constructing life tables and Kaplan–Meier survival analysis plot. The cases were grouped into 1-year intervals, with failure defined as revision to TKR or need for revision. The mean survivorship of the implant was 96 % at 10.9 years with 95 % CI (10.6, 11.4 years) (Fig. 5).

## Discussion

The long-term success of UKR depends on stringent patient selection criteria and surgical technique. The benefits of UKR over TKR are better ROM, faster recovery and greater patient satisfaction [11–13]. Reports have shown that UKR continues to give a result as good as TKR for 10–14 years [5]. The present study concluded early recovery of patient following fixed-bearing UKR, reduced hospital stay (mean 2.5 days), excellent knee ROM (118.3° ± 12°), and very good patient satisfaction (87 %). The blood loss was minimal and no patient required blood transfusion following the UKR procedure.

Anterior knee pain and medial facet PF arthritis will improve following UKR [16–19]. In our series, 105 patients (60 %) with medial facet PF arthritis had no progression of PF arthritis or had anterior knee joint pain during the follow-up. Patients with lateral facet PF arthritis and mal-tracking were not included in the study.

Loosening of a component, progression of arthritis to the retained compartments, and polyethylene wear, were three major causes of failure in UKR. In the Swedish Knee Arthroplasty Register (SKAR) 2011 [20], the most common cause of failure of UKR was loosening of a component and approximately 45 % of revisions were attributed to this cause from 2000−2009. Lewold et al. [21] reported risk of revision following Oxford UKR was 2.1 % and mean time of revision was 26 months (range 6–74 months). In our series, two knees (1.14 %) were revised to TKR for tibial component loosening and mean time of revision was 6 years (range 5–7 years). The KSS knee and functional scores following the revision surgery were satisfactory and average knee ROM was 110° (range 100°–120°).

Polyethylene wear has often been cited as a cause of failure after UKR, more so in association with flat articulating surfaces than with congruent mobile bearings [19, 21–23]. Berger et al. [24] reported no revisions for polyethylene wear in a series of 51 knees that were treated with flat articulating surface (fixed-bearing Miller−Galante). In the SKAR Annual Report 2011 [20], polyethylene wear was the second most common cause of failure of UKR and 15 % of revision cases were attributed to this cause from 2000−2009.

In our series, one knee (0.5 %) was revised to TKR for polyethylene wear at 9.6 years following primary UKR. No significant osteolysis or implant loosening was noted during the surgery.

The other main cause of failure was unexplained pain which persists even after UKR. The possible explanations include tibial condyle overload, overhang of the tibial component, overstretching of the medial collateral ligament (bearing is too thick) and pes anserine bursitis. Revision arthroplasty does not cure pain in every case, and in SKAR 2004 [34] unexplained pain following UKRs was reported to be 5–6 %. Such procedures are often not only unnecessary but also ineffective [23]. In our study, four patients (2.28 %) were revised to TKR at an average of 31.7 months (range 19–54 months) for persistent pain. There was no improvement in function or pain following revision surgery and average KS knee and functional scores were 59.5 and 66.2, respectively. Patients were not satisfied following the revision procedure and continued to complain of pain.

One patient developed chronic regional pain syndrome following UKR. TKR was not offered as the next therapeutic step because it worsens the condition which was managed symptomatically.

Most authors reported that overcorrection of the varus deformity into valgus deformity is the usual cause for progression of arthritis in the contralateral compartment and recommend leaving the UKR knee in a few degrees of varus or neutral to avoid this [21–24, 40]. In the SKAR 2004 Report [34], approximately 25 % of the UKR revisions were for progression of arthritis. Progression of arthritis in other compartments, either PF or lateral, was not encountered in our study. This was attributed to slight undercorrection or neutral correction of the deformity and the mean polyethylene thickness used was 9 mm (range 8–12 mm).The mean post-operative alignment of the knees was 3° (range neutral to 5°) and at recent follow-up was 4° (range 2°–8°).

Studies have reported that TKR patients with a high BMI have inferior results compared to patients with a lower BMI [24–30]. Tabor et al. [31] reported in a mean follow-up of 20 years in 82 patients that obese patients had higher survival than those who were not obese. In another study of patients with Oxford Phase III UKR, Kuipers et al. [32] reported no early difference between obese and non-obese patients. In our series, there was no statistical significant difference in the clinical and functional outcome following UKRs in the cohort of BMI ≥30 and ≤30 kg/m^2^ (*p* > 0.05).

Lidgren et al. [34] reported age at the time of surgery to be a recognised risk factor for implant survival both in UKR and TKR. Reliable function and good survival have been reported for TKR in younger patients, and this form of treatment has also been advocated for unicompartmental osteoarthritis [31, 33–38]. The advantages of UKR over TKR include retention of the cruciate ligaments, preservation of bone stock in the uninvolved compartments and better functional results [34, 39, 40]. In our study cohort, 39 (21.7 %) were aged ≤55 years and 137 (78.3 %) were aged ≥55 years. There was no statistically significant difference in KS knee and functional scores between the group aged ≤55 years and those aged ≥55 years.

Voss et al. [26] also reported that most patients with UKR walked with a normal gait pattern as assessed in the gait laboratory. Rourgraff et al. [14] reported better clinical results and prosthetic survivorship for UKR over TKR and also reported that more people preferred UKR. In our study cohort, 87 % of the patients showed good to excellent functional and clinical outcome, and preferred UKR for the opposite knee. Twelve patients in our study who had TKR in the other knee preferred UKR over TKR. Most patients had near normal knee kinematics and were happy with the gait pattern following UKR.

Implant survival was calculated by constructing life tables and Kaplan–Meir survival analysis plot. The cases were grouped into 1-year intervals, with failure defined as revision to TKR or need for revision. The mean survivorship of the implant was 96 % at 10.9 years with 95 % CI (10.6, 11.4 years) in our study group, which was comparable to other published studies [6, 22, 41].

The main limitation of our study was that it was a non-randomized case series study (single surgeon) and the results were not compared with a controlled group. Patients and their respective clinical and functional results were not matched based on age, BMI and pre-operative limb alignment. The average follow-up of the study was short when compared with the most series in the literature.

The study concludes that fixed-bearing Miller−Galante UKR is a valid alternative for patients with idiopathic medial compartment knee arthritis and the learning curve is steep. Proper patient selection is the key for excellent functional outcome and high rate of survivorship of implant following UKR.

## References

[1] Peat G, Mc carney R, Croftt P (2001). Knee pain and osteoarthritis in older adults; a review of community burden and current use of health care. Ann Rheum Dis.

[2] Hootman JM, Helmick CG (2006). Projections of US prevalence of arthritis and associated activities limitations. Arthritis Rheum.

[3] American College of Rheumatology Subcommittee on Osteoarthritis Guidelines (2000). Recommendations for the medical management of osteoarthritis of the hip and knee. Arthritis Rheum.

[4] Ahlback S (1968). Osteoarthrosis of the knee: a radiographic investigation. Acta Radiol Diagn (Stockh).

[5] Fisher N, Agarwal M, Reuben SF, Johnson DS, Turner PG (2006). Sporting and physical activity following Oxford medial unicompartmental knee arthroplasty. Knee.

[6] Price AJ, Waite JC, Svard UC (2005). Long term clinical results of the medial oxford unicompartmental knee arthroplasty. Clin Orthop.

[7] Bedson J, Jordon K, Croft P (2004). The prevalence and history of knee osteoarthritis in general practice: a case-control study. Fam Pract.

[8] Murray DV, Goodfellow JW, O’Connor JJ (1998). The Oxford medial unicompartmental arthroplasty: a 10-year survival study. J Bone Joint Surg Br.

[9] Bert JM (2005). Unicompartmental knee replacement. Orthop Clin N Am.

[10] Laurencin CT, Zelicof SB, Scott RD (1991). Unicompartmental versus total knee arthroplasty in the same patient. A comparative study. Clin Orthop.

[11] Svard UCG, Price AJ (2001). Oxford medial unicompartmental knee arthroplasty. A survival analysis of an independent series. J Bone Joint Surg Br.

[12] Tanavalee A, Choi YJ, Tria AJ (2005). Unicondylar knee arthroplasty: past and present. Orthopedics.

[13] Ackroyd CE, Whitehouse SL, Newman JH, Joslin CC (2002). A comparative study of the medial St Georg sled and kinematic total knee arthroplasties. Ten-year survivorship. J Bone Joint Surg Br.

[14] Berger RA, Meneghini RM, Jacobs JJ (2005). Results of unicompartmental knee arthroplasty at a minimum of 10 years of follow-up. J Bone Joint Surg Am.

[15] BrennerSmith At, Ewings P, Weale AE (2004). Knee scores in a normal elderly population. Knee.

[16] Rougraff BT, Heck DA, Gibson AE (1991). A comparison of tricompartmental and unicompartmental arthroplasty for the treatment of gonarthrosis. Clin Orthop.

[17] Jonsson GT (1981) Compartmental arthroplasty for gonarthrosis. Acta Orthop Scand Suppl 193 10.3109/17453678109154867 6243053

[18] Goodfellow Hungerford DS, Zindel M (1976). Patella-femoral joint mechanics and pathology. 1. Functional anatomy of the patella-femoral joint. J Bone Joint Surg Br.

[19] Beard DJ, Pandit H, Ostlere S, Jenkins C, Dodd CAF, Murray DW (2007). Pre-operative clinical and radiological assessment of the patellofemoral joint in unicompartmental knee replacement and its influence on outcome. J Bone Joint Surg Br.

[20] Sundberg M, Lidgren L, Knutson K, Robertson O. Swedish Knee Arthroplasty Register 2011 annual report. http://www.myknee.se/en

[21] Lewold S, Goodman S, Knutson K, Robertson O, Lidgren I (1995). Oxford meniscal bearing knee versus the Marmor knee in unicompartmental arthroplasty for arthrosis. A Swedish multicentre survival study. J Arthroplasty.

[22] Argenson Jn, O’Connor JJ (1992). Polyethylene wear in meniscal knee replacement. A one to nine-year retrieval analysis of the Oxford knee. J Bone Joint Surg Br.

[23] Psychoylos V, Crawford RW, O’Connor JJ, Murray DW (1998). Wear of congruent meniscal bearings in unicompartmental knee replacement: a retrieval study of 16 specimens. J Bone Joint Surg Br.

[24] Berger RA, Nedeff DD, Barden RM, Sheinkop MM, Jacobs JJ, Rosenberg AG, Galante JO (1999). Unicompartmental total knee arthroplasty. Clinical experience at 6- to10-year follow up. Clin Orthop.

[25] Squire MW, Callaghan JJ, Goetz DD, Sullivan PM, Johnston RC (1999). Unicompartmental knee replacement. A minimum 15 year follow up study. Clin Orthop.

[26] Voss F, Sheinkop MB, Galante JO, Barden RM, Rosenberg AG (1995). Miller-Galante unicompartmental knee arthroplasty at 2- to 5-year follow-up evaluation. J Arthroplasty.

[27] Foran JR, Mont MA, Rajadhyaksha AD (2004). Total knee arthroplasty in obese patients: a comparison with a matched control group. J Arthroplasty.

[28] Winiarsky R, Barth P, Lotke P (1998). Total knee arthroplasty in morbidly obese patients. J Bone Joint Surg Am.

[29] Berend KR, Lombardi AV, Mallory TH (2005). Early failure of minimally invasive unicompartmental knee arthroplasty is associated with obesity. Clin Orthop Relat Res.

[30] Heck DA, Marmor L, Gibson A (1993). Unicompartmental knee arthroplasty. A multicentre investigation with long term follow up evaluation. Clin Orthop Relat Res.

[31] Tabor OB, Tabor OB, Bernard M (2005). Unicompartmental knee arthroplasty: long term success in middle age and obese patients. J Surg Orthop Adv.

[32] Kuipers BM, Kollen BJ, Kaijser Bots PC (2010). Factors associated with reduced early survival in the Oxford phase III medial unicompartmental knee replacement. Knee.

[33] Peter MB, Maria SG, Micael GZ, Harpal SK, Aaron JJ, Michael AM (2011). Outcomes of unicompartmental knee arthroplasty stratified by body mass index. J Arthroplasty.

[34] Lidgren L, Knutson K, Robertson O. Swedish Knee arthroplasty register 2004 annual report. www.myknee.se/en

[35] Argenson JN, Chevrol-Bankeddache Y, Aubaniac JM (2002). Modern unicompartmental knee arthroplasty with cement: a three to 10-year follow-up study. J Bone Joint Surg Am.

[36] Price AJ, Dodd CA, Svard UCG, Murray DW (2005). Oxford medial unicompartmental knee arthroplasty in patients younger and older than 60 years of age. J Bone Joint Surg Br.

[37] Pennington DW, Swienchowski JJ, Lutes WB, Drake GN (2003). Unicompartmental knee arthroplasty in patients 60 years of age or younger. J Bone Joint Surg Am.

[38] Parratte S, Argenson JNA, Pearce O, Pauly V, Auquier P, Aubaniac JM (2009). Medial unicompartmental knee replacement in the under-50 s. J Bone Joint Surg Br.

[39] Hanssen AD, Stuart MJ, Scott RD, Scuderi GR (2001). Surgical options for the middle aged patient with osteoarthritis of the knee joint. Instr Course Lect.

[40] Berger RA, Meneghini RM, Sheinkop MB, Della Valle CJ, Jacobs JJ, Rosenberg AG, Galante JO (2004). The progression of patellofemoral arthrosis after medial unicompartmental replacement: results at 11 to 15 years. Clin Orthop Rel Res.

[41] Weale AE, Newman JH (1994). Unicompartmental arthroplasty and high tibia osteotomy for osteoarthrosis of the knee: a comparative study with a 12- to 17-year follow-up period. Clin Orthop.

